# Characterization and neurogenic responses of primary and immortalized Müller glia

**DOI:** 10.3389/fcell.2025.1513163

**Published:** 2025-05-09

**Authors:** Thi-Hang Tran, Donny Lukmanto, Mei Chen, Olaf Strauß, Toshiharu Yamashita, Osamu Ohneda, Shinichi Fukuda

**Affiliations:** ^1^ Laboratory of Advanced Vision Science, Institute of Medicine, University of Tsukuba, Tsukuba, Japan; ^2^ Laboratory of Regenerative Medicine and Stem Cell Biology, Institute of Medicine, University of Tsukuba, Tsukuba, Japan; ^3^ Ph.D. program in Human Biology, School of Integrative and Global Majors, University of Tsukuba, Tsukuba, Japan; ^4^ The Wellcome-Wolfson Institute for Experimental Medicine, School of Medicine, Dentistry and Biomedical Sciences, Queen’s University Belfast, Belfast, United Kingdom; ^5^ Experimental Ophthalmology, Department of Ophthalmology, Charité - Universitätsmedizin Berlin, Corporate Member of Freie Universität, Berlin Institute of Health, Humboldt-University, Berlin, Germany

**Keywords:** Müller glia, QMMuC-1 cell line, ImM10 cell line, neurogenic responses, chemical induction, neuronal reprogramming

## Abstract

Primary Müller glia (MG) have been reported to exhibit a neurogenic capacity induced by small molecules. However, whether immortalized mouse MG cell lines exhibit neurogenic capacities similar to those of primary mouse MG remains unclear. In this study, we examined the morphology, proliferation rate, and marker profile of primary MG cells isolated from postnatal mouse pups with two immortalized mouse MG cell lines, QMMuC-1 and ImM10, in a standard growth medium. After chemical induction, we compared the morphology, markers, direct neuronal reprogramming efficiency, and axon length of these cell types in two culture media: Neurobasal and DMEM/F12. Our results showed that in standard growth medium, QMMuC-1 and ImM10 cells displayed similar morphology and marker profiles as primary MG cells, with the only differences observed in nestin expression. However, QMMuC-1 and ImM10 cells exhibited much higher proliferation rates than the primary MG cells. Following chemical treatment in both Neurobasal and DMEM/F12 media, a subset of primary MG, QMMuC-1, and ImM10 cells was induced to differentiate into immature neuron-like cells by day 7. While primary MG cells showed similar neuronal reprogramming efficiency and axon length extension in both media, QMMuC-1 and ImM10 cells displayed variations between the two culture media. Moreover, some of the induced neuronal cells derived from primary MG cells expressed HuC/D and Calbindin markers, whereas none of the cells derived from QMMuC-1 and ImM10 cells expressed these markers. Subsequent observations revealed that induced immature neuron-like cells derived from primary MG cells in both types of media and those derived from ImM10 cells cultured in DMEM/F12 survived until day 14. Taken together, our findings suggest that the two immortalized cell lines, QMMuC-1 and ImM10, exhibited neurogenic capacities similar to those of primary MG cells to some extent but did not fully recapitulate all their characteristics. Therefore, careful consideration should be given to culture conditions and the validation of key results when using immortalized cells as a substitute for primary MG cells.

## 1 Introduction

Many retinal degenerative diseases are characterized by the progressive degeneration of retinal neurons, including photoreceptors and retinal ganglion cells, which eventually leads to irreversible blindness (Kaur and Singh, 2023). Cell replacement is a promising strategy for replacing lost neurons and restoring vision (Coco-Martin et al., 2021). However, cell replacement through transplantation poses several challenges in clinical applications, including the complicated process of creating a reliable cell source, poor survival and integration rates of cells, and other potential side effects (Coco-Martin et al., 2021). In contrast, cell replacement via *in vivo* direct reprogramming from an endogenous cell source to neurons can overcome these challenges (Coco-Martin et al., 2021; Wang et al., 2021). Direct reprogramming of resident cells to replace damaged neurons is faster and avoids the risk of immune rejection (Wang et al., 2021). Nonetheless, this strategy requires further investigation to address significant limitations such as the low proliferative ability of the endogenous cell source, low conversion efficiency, and immaturity of the converted neurons (Wang et al., 2021). More importantly, several studies that successfully achieved *in vivo* direct reprogramming lack definitive validation of the origin of the converted neurons, leading to controversy or issues with reproducibility (Yao et al., 2018; Zhou et al., 2020; Xu et al., 2023). To address this limitation, genetic lineage tracing of the starting cells involved in direct reprogramming should be used to ensure the accurate identification and validation of the reprogrammed neurons (Hoang et al., 2022; Xie and Chen, 2022; Xie et al., 2022).

Several studies have successfully converted Müller glia (MG), the radial glia in the retina, into retinal neurons by overexpressing neuronal transcription factor genes in adult mice (Jorstad et al., 2017; Yao et al., 2018; Todd et al., 2021; Todd et al., 2022). However, for clinical use, inducing neuronal conversion using small molecules is desirable because this approach is cost-effective, safe, and easy to control in terms of concentration and timing (Wang et al., 2021). Two studies showed that chemical compounds can convert primary MG cells isolated from rat and mouse pups into bipolar-like cells (Xia et al., 2021; Yang et al., 2022). Interestingly, one study found that the intravitreal injection of four small molecules induced some MG to migrate into the outer nuclear layer and express rhodopsin, a specific gene of rod photoreceptors (Fujii et al., 2023). Despite these encouraging findings, the efficiency of chemically induced direct reprogramming remains low, and it is not yet understood how small-molecule combinations can be adjusted to achieve the desired retinal neurons.

In 2015, through two successive rounds of chemical screening with 5,000 and 1,500 small molecules, a set of five small molecules was found to effectively induce mouse fibroblasts into functional neurons *in vitro* (Li et al., 2015). This compound combination was optimized to convert astrocytes into neurons in the mouse brain (Ma et al., 2021). Thus, *in vitro* implementation offers many advantages for screening drug candidates owing to its cost-effectiveness and rapid procedures. Nonetheless, to obtain a pure primary MG population for *in vitro* experiments, retinas are usually dissociated using several enzymes, cultured, and passaged at least twice (Liu et al., 2017; Pereiro et al., 2020b). Using this method, only a small number of primary MG cells can be obtained; however, they proliferate slowly and early undergo senescence after four to eight passages (Liu et al., 2017; Pereiro et al., 2020b). Thus, it requires the use of many postnatal mouse pups to obtain enough cells for experiments, which raises ethical concerns regarding animal welfare. Several MG cell lines have been established and used to study the characteristics and functions of MG, owing to their high proliferative capacity (Sarthy et al., 1998; Limb et al., 2002; Tomi et al., 2003; Otteson and Joseph Phillips, 2010; Augustine et al., 2018; Kittipassorn et al., 2019). Despite the similar characteristics of these cell lines to those of primary MG cells, the immortalization process may change their physiology and behavior compared to primary cells. However, it remains unknown whether these cell lines can serve as useful tools for studying neurogenic characteristics instead of primary MG cells. Here, we show similar and distinct characteristics between primary MG, ImM10, and QMMuC-1 cells grown in growth and chemical induction media. These findings provide useful information on the use of primary MG or immortalized cells to study the neurogenic capacity of MG.

## 2 Materials and methods

### 2.1 Experimental animals

Wild-type C57BL/6J female and male mice were purchased from CLEA Japan Inc (Tokyo, Japan). The mice were kept at the laboratory of the Animal Resource Center facility of the University of Tsukuba under standard conditions, including controlled temperatures (23°C ± 1°C) and a 12-h light/dark cycle (7 a.m.–7 p.m.). The mice had *ad libitum* access to water and chow. The animal experimental procedure were reviewed and approved by the Animal Experimental Ethical Review Committee of the University of Tsukuba.

### 2.2 Isolation and culture of Primary Müller glia

Primary MG were isolated from the retinas of postnatal day 2 C57BL/6J mouse pups using a Papain Dissociation System (Worthington Biochemical, Cat. #LK003150), following previously established protocols with some modifications (Liu et al., 2017). Briefly, retinas were dissected from the eyes and then incubated with Papain for 10 min in a water bath at 37°C. Every 5 min, the tubes containing the dissociated solutions were gently shaken by rotating them up and down several times. Once incubation was completed, ovomucoid and DNase I were added to the tissue solution to halt the dissociation process. The cell suspension was centrifuged at 400 *g* for 5 min at room temperature. Subsequently, the cell pellet was washed and resuspended in high-glucose DMEM medium (Gibco, Cat. #12100061) supplemented with 10% Fetal Bovine Serum (FBS; Gibco, Cat. #10270-106) and 1% penicillin-streptomycin (Fujifilm Wako Pure Chemical, Cat. #168-23191), followed by centrifugation at 300 *g* for 5 min at room temperature. Finally, the cell pellet was resuspended in in high-glucose DMEM medium supplemented with 10% FBS and 1% penicillin-streptomycin and seeded onto 0.1% gelatin-coated dishes. After two passages, the primary cells were utilized for characterization and chemical reprogramming.

### 2.3 Culture of two immortalized müller glia cell lines

The QMMuC-1 cell line was generously provided by Dr. Mei Chen, Queen’s University Belfast. The ImM10 cell line was kindly provided by Dr. Olaf Strauß, Charité Universitäts Medizin Berlin, with approval from Dr. Deborah C. Ottenson, University of Houston. Both cell lines were cultured in high-glucose DMEM medium supplemented with 10% FBS and 1% penicillin-streptomycin. QMMuC-1 and ImM10 cells at passages 30-40 were used in this study.

### 2.4 Proliferation rate assay

The proliferation rates of primary MG, QMMuC-1 and ImM10 cells were determined using the Cell Counting Kit 8 (Dojindo, Japan) following the manufacturer’s instructions. Initially, primary MG, QMMuC-1 and ImM10 cells were seeded in 96-well plates at a density of 1 × 10^3^ cells/well and cultured in high-glucose DMEM supplemented with 10% FBS and 1% penicillin/streptomycin. Cell proliferation was measured every 24 h for 7 days. At the designated time point, 10 μL of Cell Counting Kit 8 reagent was added to each well and mixed uniformly. All cells were incubated for an hour, and the optical absorbance at 450 nm was measured and averaged from six wells for each cell type using a Varioskan Lux multiplate reader (Thermo Fisher Scientific, United States).

### 2.5 Small-molecule-induced direct reprogramming of primary and immortalized MG

Primary MG, QMMuC-1, and Imm10 cells were chemically reprogrammed according to a previously reported method with some modifications (Yang et al., 2022). Briefly, primary MG, ImM10, and QMMuC-1 cells were seeded into 96-well plates coated with 2% Matrigel (Corning, Cat. #354234) and cultured in a growth medium. After primary MG and QMMuC-1 cells reached over 90% confluence and ImM10 cells reached 70% confluence, cells were changed into neuronal induction medium (100 μL of medium per well), consisting of Neurobasal (Gibco, Cat. #21103-049) or DMEM/F12 medium (Gibco, Cat. #11320-033) supplemented with 0.5% N2 (Thermo Fisher Scientific, Cat. #17502048), and 1% B27 (Thermo Fisher Scientific, Cat. #17504,044), 1% L-glutamine (Gibco, Cat. #25030081), 1% penicillin-streptomycin, basic fibroblast growth factor (100 ng/mL; Peprotech, Cat. #100-18B) (100 ng/mL), cAMP (100 μM; Nacalai Tesque, Cat. #23840-16), forskolin (10 μM; Sigma-Aldrich, Cat. #F6886), ISX9 (40 μM; Selleck Chemicals, Cat. #1300031-49-5), CHIR99021 (20 μM; AdooQ BioScience, Cat. #A10199), I-BET151 (2 μM; Medchem Express, Cat. #HY-12323), and Y-27632 (5 μM; Merck Millipore, Cat. #688001). On day 2 after chemical treatment, the concentrations of three small molecules were reduced (ISX9, 20 μM; CHIR99021, 3 μM; I-BET151, 1 μM). On day 7 after chemical induction, the cells were fixed and subjected to immunofluorescence analysis.

For the control groups, only DMSO or individual small molecules were added to the medium. Specifically, ISX9 (day 0 to day 2: 40 μM, day 2 to day 7: 20 μM) or I-BET151 (day 0 to day 2: 2 μM, day 2 to day 7: 1 μM) was used as a control treatment.

### 2.6 Immunofluorescence staining

The cells were fixed in 4% paraformaldehyde for 20 min at room temperature, followed by three washes with PBS, each lasting 5 min. Subsequently, the cells were permeabilized with 0.3% Triton in PBS for 10 min. After incubation with a blocking buffer containing 1% bovine serum albumin (Sigma-Aldrich, Cat. #9048-46-8) and 0.3% Triton in PBS for 1 h, the cells were incubated with primary antibodies diluted in the blocking buffer overnight at 4°C. The cells were washed with 0.05% Tween in PBS three times, each for 5 min before incubation with secondary antibodies and 4′,6-diamino-2-phenylindole (DAPI; Invitrogen Corporation, Carlsbad, CA; 1:1,000 dilution) diluted in the blocking buffer for 1 h at room temperature. After another round of washing with 0.05% Tween in PBS three times for 5 min each, the cells were mounted with non-hardening Fluoro-KEEPER Antifade Reagent (Nacalai Tesque, Japan) and imaged using a Keyence BZ-X700 All-in-One microscope (Keyence, Osaka, Japan).

The primary antibodies used in this study were rabbit sex determining region Y (SRY)-box9 (Sox9; Merck, Cat. #AB5535, 1:100), rabbit glutamine synthetase (GS; Abcam, Cat. #ab73593, 1:100), rabbit glutamate aspartate transporter (GLAST; Frontier Institute, Cat. #GLAST-Rb-Af660, 1:100), rabbit glial fibrillary acidic protein (GFAP; CST, Cat. #12389, 1:100), mouse nestin (Invitrogen, Cat. #14-5,843-82, 1:500), rabbit paired box 6 (Pax6; BioLegend, Cat. #BL-901301, 1:300), mouse class III beta-tubulin (TuJ1; Genetex, GT11710, 1:500), rabbit RNA-binding protein with multiple splicing (Rbpms; Abcam, Cat. # Ab194213, 1:200), rabbit Brn3a (Abcam, Cat. # Ab245230, 1:500), rabbit Calbindin (Proteintech, Cat. # 14479-1-AP, 1:200), mouse Rhodopsin (Abcam, Cat. # ab3267, 1:200), rabbit microtubule-associated protein 2 (Map2; EMD Millipore, Cat. # AB5622, 1:200), mouse HuC/D (Santa Cruz Biotechnology, Cat. # sc-515624, 1:200), rabbit NeuroD1 (CST, Cat. #D90G12-7,019, 1:200), and rabbit Otx2 (PGI, Cat. #13497-1-AP, 1: 500).

The secondary antibodies used in the present study included Alexa Fluor® 488 goat anti-rabbit IgG H&L (Abcam, Cat. #ab150077) and Alexa Fluor® 594 goat anti-mouse IgG H&L (Abcam, Cat. #ab150116) at a 1:200 dilution.

### 2.7 Quantification and statistical analysis

The fluorescence intensity was measured using NIH ImageJ/Fiji software, following a previously published method with some modifications (Echeverria et al., 2025). Images taken under the same settings across cell types were converted to grayscale. The freehand tool was used to isolate individual cells, and features such as the “area,” “area of integrated intensity,” and “mean gray value” were measured. A similar area was drawn in a region without cells to measure the background fluorescence intensity. The corrected total cell fluorescence for each cell was calculated by subtracting the product of the “mean gray value” of the background and the “area” of each single cell from the “area integrated density” of that cell. To account for differences in cell size, the corrected total cell fluorescence was normalized by dividing it by the “area” of the respective cell to obtain the fluorescence intensity ratio. The fluorescence intensity ratio of the three cell types in the growth medium was assessed using 3 to 5 cells per image, with 15 images analyzed for each cell line. The fluorescence intensity ratio of TuJ1 at day 14 was evaluated for 1 to 5 cells per image, with 5–10 images analyzed for each cell line.

The conversion efficiency and axon length were analyzed using the Simple Neurite Tracer plugin in the NIH ImageJ/Fiji software (Arshadi et al., 2021). For each treatment, 10-15 images at ×20 magnification were analyzed to obtain average values. Three independent experiments were conducted.

The percentages of primary MG cells positive for Calbindin/TuJ1 or HuC/D among total cells were quantified on day 7 after chemical treatment, using 15 images captured at ×20 magnification.

Data processing and analyses were performed using Python (version 3.8). Statistical significance was determined using the Kruskal–Wallis test, followed by Dunn’s *post hoc* test for multiple comparisons (*p < 0.05).

## 3 Results

### 3.1 Cellular morphology

**FIGURE 1:**
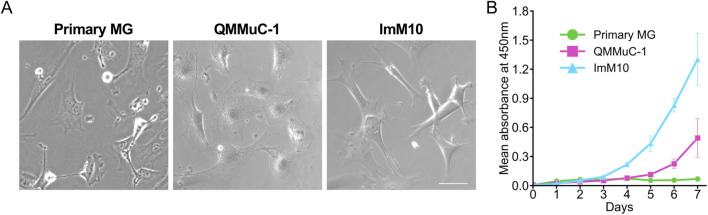
Morphology and proliferation rate of primary MG, QMMuC-1, and ImM10 cells in growth media. **(A)** The cellular morphology of primary MG, QMMuC-1, and ImM10 cells was observed under light microscopy. Scale Bars, 50 μm. **(B)** The proliferation rate of primary MG, QMMuC-1, and ImM10 cells in growth media for 7 days. Both immortalized cell lines exhibited a higher proliferation rate than primary MG. Error bars show mean ± SD.

After isolation and two passages, the cellular morphology of primary MG was examined and compared with that of QMMuC-1 and ImM10 cells under light microscopy (Figure 1A). All cell cultures exhibited a typical MG morphology, with large adherent cells and multiple broad processes. However, the ImM10 cells showed a slight difference in morphology, with elongated rather than broad processes.

### 3.2 Proliferation rate

The proliferation rates of primary MG, QMMuC-1, and ImM10 cells at 1 week were determined using a Cell Counting Kit 8 (Figure 1B). For the first 3 days, all cell types showed comparable proliferation rates. However, from day 3, ImM10 cells proliferated remarkably, and on day 7, the mean absorbance at 450 nm of ImM10 cells was approximately 19 times and 2.65 times greater than that of primary MG and QMMuC-1 cells, respectively. The proliferation rate of QMMuC-1 cells increased gradually, with the mean absorbance at day 7 being 7.3 times higher than that of primary MG cells. In contrast, primary MG cells only proliferated for the first 2 days and then maintained a similar number of cells until day 7. This suggests that QMMuC-1 and ImM10 cells have a higher proliferative capacity than primary MG cells, as expected.

### 3.3 Cellular marker profile

**FIGURE 2:**
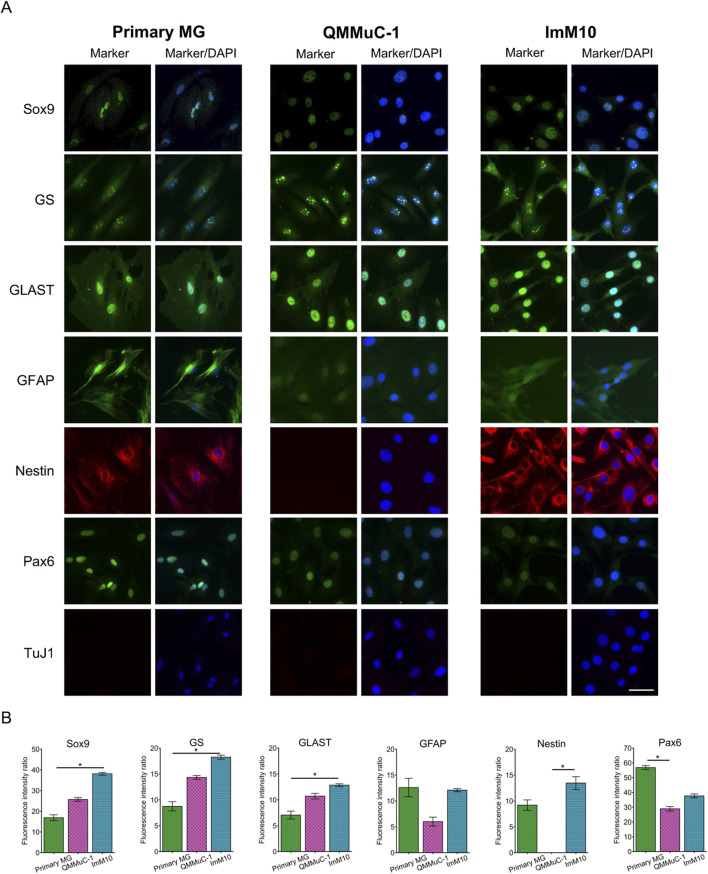
Marker profile of primary MG, QMMuC-1, and ImM10 cells in growth media. **(A)** Representative images of primary MG, QMMuC-1, and ImM10 cells stained with MG markers (Sox9, GS, GLAST, and GFAP), retinal progenitor markers (nestin, Pax6), and a neuronal marker (TuJ1). Scale Bars, 50 μm. **(B)** The fluorescence intensity ratio for each marker. The data are presented as mean ± SD.

We investigated the expression of primary MG, QMMuC-1, and ImM10 cells with various markers, including marker specific for MG, neuronal progenitor cells, and neurons by immunocytochemistry (Figure 2). All cell cultures expressed three MG markers: sex-determining region Y-box 9 (Sox9), glutamine synthetase (GS), and glutamate aspartate transporter (GLAST). ImM10 cells exhibited significantly higher fluorescence signal intensities for all three markers compared to primary MG. Glial fibrillary acidic protein (GFAP), a marker of MG in the activated state, especially after retinal damage, was expressed by all three cell types. However, primary MG showed higher variability in GFAP expression, indicating heterogeneous characteristics.

Interestingly, both primary MG and ImM10 cells were positive for a neuronal progenitor cell marker nestin, whereas QMMuC-1 cells were negative. All three cell types expressed Pax6. None of the primary MG, QMMuC-1, or ImM10 cells expressed class III beta-tubulin (TuJ1), a marker of immature neurons. These results suggest that QMMuC-1 and ImM10 cells exhibit a marker profile highly similar to that of primary MG cells, except for nestin marker, with some variation in fluorescence signal intensity.

### 3.4 Morphological change and marker expression during chemical reprogramming

**FIGURE 3:**
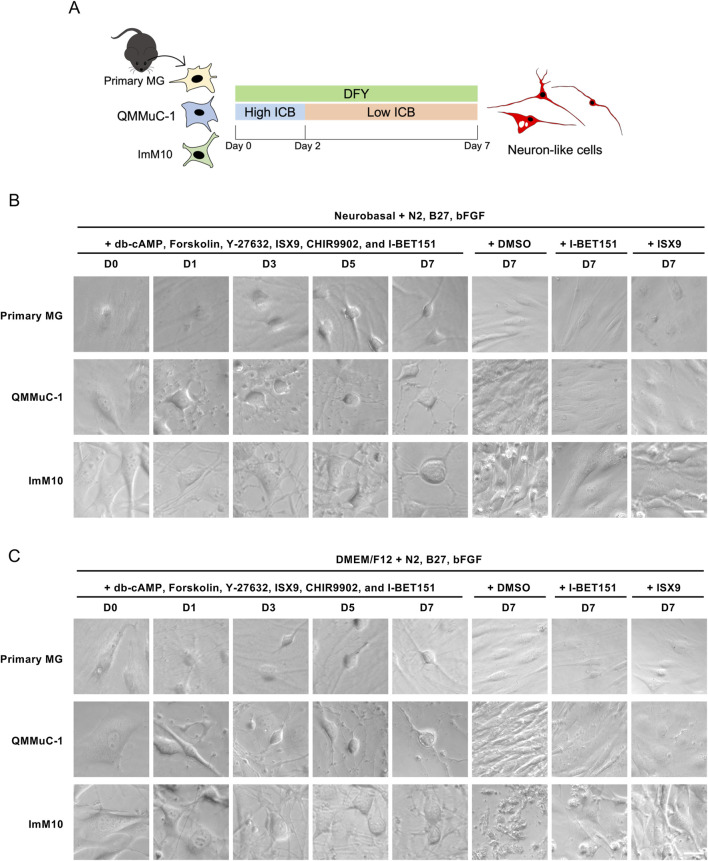
Morphological changes of primary MG, QMMuC-1, and ImM10 cells after chemical treatment. **(A)** The scheme of the chemical induction procedure. Primary MG, QMMuC-1, and ImM10 cells were seeded onto matrigel-coated wells and then cultured with an induction medium consisting of db-cAMP (D, 100 μM), Forskolin (F, 10 μM), Y-27632 (Y, 5 μM), ISX9 (I, 40 μM), CHIR99021 (C, 20 μM), and I-BET151 (B, 2 μM). After 2 days, the concentrations of three compounds were reduced (ISX9, 20 μM; CHIR99021, 3 μM; I-BET151, 1 μM). **(B)** Phase-contrast images of cells on days 0, 1, 3, 5, and 7 in a Neurobasal medium supplemented with 6 small molecules and the phase-contrast images of cells on days 7 in a Neurobasal medium supplemented with DMSO or only I-BET151 or only ISX9. **(C)** Phase-contrast images of cells on days 0, 1, 3, 5, and 7 in DMEM/F12 medium and the phase-contrast images of cells on days 7 in a DMEM/F12 medium supplemented with DMSO or only I-BET151 or only ISX9. Primary MG, QMMuC-1, and ImM10 cells changed their cell shape from glial morphology to neuron-like morphology during the chemical induction process in both media supplemented with 6 compounds. Scale Bars, 25 μm.

Next, we examined whether QMMuC-1 and ImM10 cells exhibited similar neurogenic characteristics induced by small molecules as primary MG cells. We treated primary MG, QMMuC-1, and ImM10 cells with six compounds, including db-cAMP (D, 100 μM), Forskolin (F, 10 μM), Y-27632 (Y, 5 μM), ISX9 (I, 40 μM), CHIR99021 (C, 20 μM), and I-BET151 (B, 2 μM), that have been reported to induce the differentiation of primary mouse MG cells into neuron-like cells (Yang et al., 2022). Unexpectedly, we observed massive cell death during a preliminary experiment (data not shown), particularly in QMMuC-1 and ImM10 cells. Cell death is a major limiting factor in direct reprogramming (Gascón et al., 2016). A high concentration of small molecules induces rapid neuronal conversion but also causes significant cell death (Feng et al., 2021). Thus, we aimed to improve the survival rate by reducing the concentrations of three compounds on day 2 after chemical induction (Figure 3A). Additionally, we tested the neurogenic capacity of these cell cultures in two different media: Neurobasal and DMEM/F12. With this new concentration modification, we observed rapid cellular morphological changes in a subset of primary MG, QMMuC-1, and ImM10 cells after chemical treatment in both media (Figures 3B, C). A few QMMuC-1 cells acquired smaller cell bodies with thin extensions by day 3. By day 7, in both types of culture media, numerous primary MG, QMMuC-1, and ImM10 cells adopted neuron-like shapes with small cell bodies and long extensions. In contrast, in the control group with DMSO or with only IBET-151 or ISX9, all three cell types maintained typical MG-like morphology. Interestingly, in DMEM/F12 medium supplemented with DMSO, most of the ImM10 cells underwent cell death, while these cells still survived and proliferated in Neurobasal medium supplemented with DMSO. This result indicates that DMEM/F12 medium lacks certain factors or nutrients necessary for the survival and proliferation of ImM10 cells, unlike Neurobasal medium.

**FIGURE 4:**
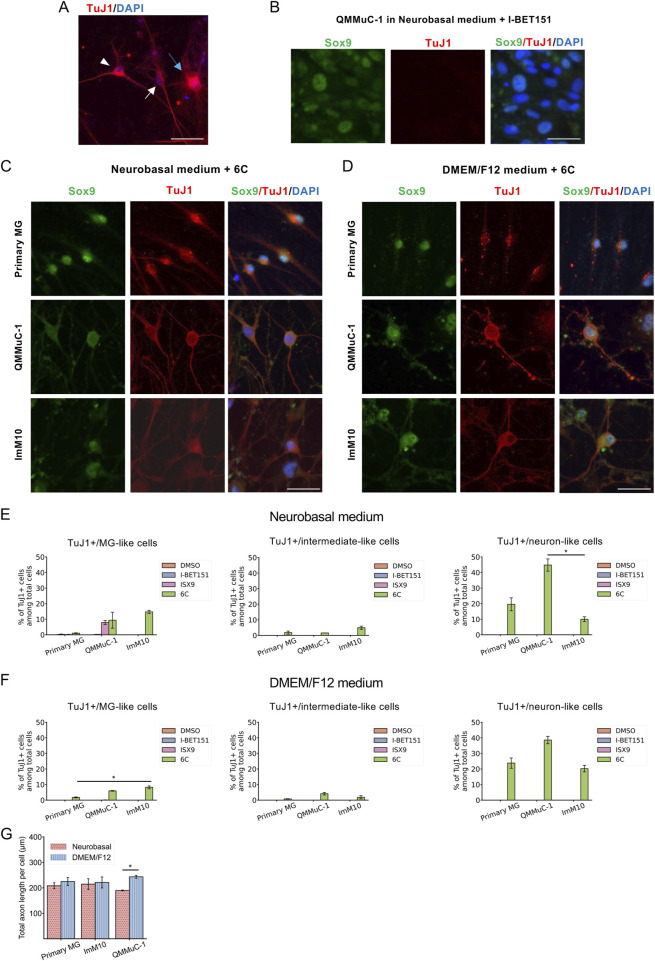
Analysis of TuJ1-positive cells derived from primary MG, QMMuC-1, and ImM10 cells at day 7 after chemical treatment. **(A)** Representative images of three morphologically distinct types of TuJ1+ cells derived from primary MG at day 7. TuJ1+ cell with neuron-like morphology is indicated by a white arrowhead. TuJ1+ cell with intermediate-like morphology is indicated by a white arrow. TuJ1+ cell with MG-like morphology is indicated by a blue arrow. Scale bars, 100 μm. **(B)** A representative image of cells in the control group that were not induced into TuJ1+ cells: QMMuC-1 cells cultured in Neurobasal medium supplemented with I-BET151 only. Scale bars, 50 μm. **(C, D)** Immunostaining of primary MG, QMMuC-1, and ImM10 cells with TuJ1 and Sox9 markers at day 7 in Neurobasal medium **(C)** and DMEM/F12 medium **(D)**. Scale bars, 50 μm. **(E–F)** The percentage of neuron-like morphology/TuJ1+ cells, intermediate-like morphology/TuJ1+ cells, and MG-like morphology/TuJ1+ cells derived from primary MG, QMMuC-1, and ImM10 cells among total number of cells at day 7 in Neurobasal medium **(E)** and DMEM/F12 medium **(F)**. The data are presented as mean ± SEM (n = 3). **(G)** The total axonal length per cell of neuron-like morphology/TuJ1+ cells at day 7. The data are presented as mean ± SEM (n = 3). 6C–Medium supplemented with 6 small molecules.

On day 7, all cell types were fixed and subjected to immunostaining with the MG marker Sox9 and the neuronal marker TuJ1 (Figure 4). As expected, many TuJ1-positive cells were detected in all cell cultures treated with the 6 chemicals. We observed three distinct types of morphologies of TuJ1-positive cells: neuron-like, intermediate-like, and MG-like shapes in all cultures of primary MG, QMMuC-1, and ImM10 cells (Figure 4A). In contrast, in the control groups, all cells were negative for the TuJ1 marker (Figure 4B and data not shown), with the exception of QMMuC-1 cells treated with only ISX9 compound in Neurobasal medium. A few TuJ1-positive cells with MG-like morphology were detected in this group. Notably, these TuJ1-positive cells still expressed Sox9 marker in their nuclei, indicating that the cells were still in an immature stage (Figures 4C, D).

We quantified the number of TuJ1-positive cells among the total number of cells for each morphological type (Figures 4E, F). The results showed that, in both Neurobasal and DMEM/F12 media supplemented with 6 small molecules, most TuJ1-positive cells derived from QMMuC-1 and primary MG cells acquired neuron-like shapes. In contrast, in the Neurobasal medium supplemented with 6 compounds, approximately half of the TuJ1-positive cells derived from ImM10 cells exhibited an MG-like shape (Figure 4E). In DMEM/F12, this rate was lower but still accounted for approximately one-third of the total number of TuJ1-positive cells (Figure 4F). Interestingly, in Neurobasal medium supplemented with only ISX9 compound, QMMuC-1 cells showed a comparable rate of TuJ1-positive cells with MG-like morphology to those in Neurobasal medium supplemented with 6 chemicals. This result suggests that ISX9, in combination with Neurobasal medium, has a modest effect on neuronal induction in QMMuC-1 cells. When comparing the neuronal conversion efficiencies of the three cell types induced by 6 small molecules (Figures 4E, F), QMMuC-1 cells showed a higher tendency to become TuJ1-positive and acquire a neuron-like shape. However, a significant difference in this rate was observed only between QMMuC-1 and ImM10 cells cultured in the Neurobasal medium. Analysis of the total axon length per cell showed that neuron-like/TuJ1-positive cells from primary MG and ImM10 cells extended to similar lengths in both media (Figure 4G). However, the axons of neuron-like TuJ1-positive cells derived from QMMuC-1 cells were shorter in the Neurobasal medium. In general, the total axonal length per cell was similar across all cell types. Thus, these results indicate that while the culture medium did not significantly affect neuronal conversion efficiency and axon growth in primary MG cells, chemical induction in DMEM/F12 medium induced more neuron-like cells in ImM10 cells and promoted better axon growth in QMMuC-1 cells than in Neurobasal medium.

**FIGURE 5:**
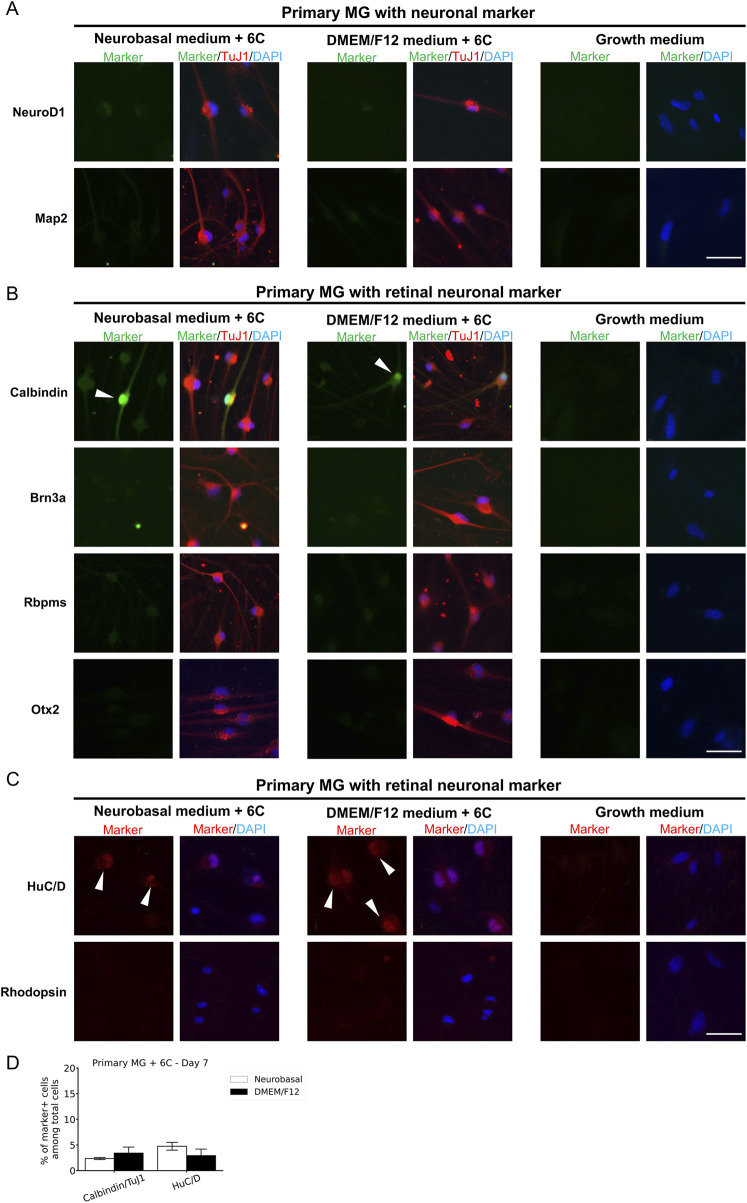
Immunostaining results of neuron-like cells derived from primary MG cells with various neuronal markers and retinal neuron markers. **(A)** Cells were stained with TuJ1 marker and neuronal markers: NeuroD1 and Map2. Scale Bars, 50 μm. **(B)** Cells were stained with TuJ1 marker and retinal neuron markers: Calbindin (primarily expressed in horizontal cell, as well as in some subtypes of amacrine and retinal ganglion cells); Brn3a and Rbpms (markers of retinal ganglion cells); Otx2 (marker of bipolar and immature photoreceptor). Scale Bars, 50 μm. **(C)** Cells were stained retinal neuron markers: HuC/D (expressed in amacrine and retinal ganglion cells, with transient expression in horizontal cells during development), Rhodopsin (marker of mature rod photoreceptor). **(D)** The percentage of cells derived from primary MG that were positive for Calbindin/TuJ1 or HuC/D among total cells on day 7. The data are presented as mean ± SD (n = 3). Scale Bars, 50 μm. 6C–Medium supplemented with 6 small molecules.

**FIGURE 6:**
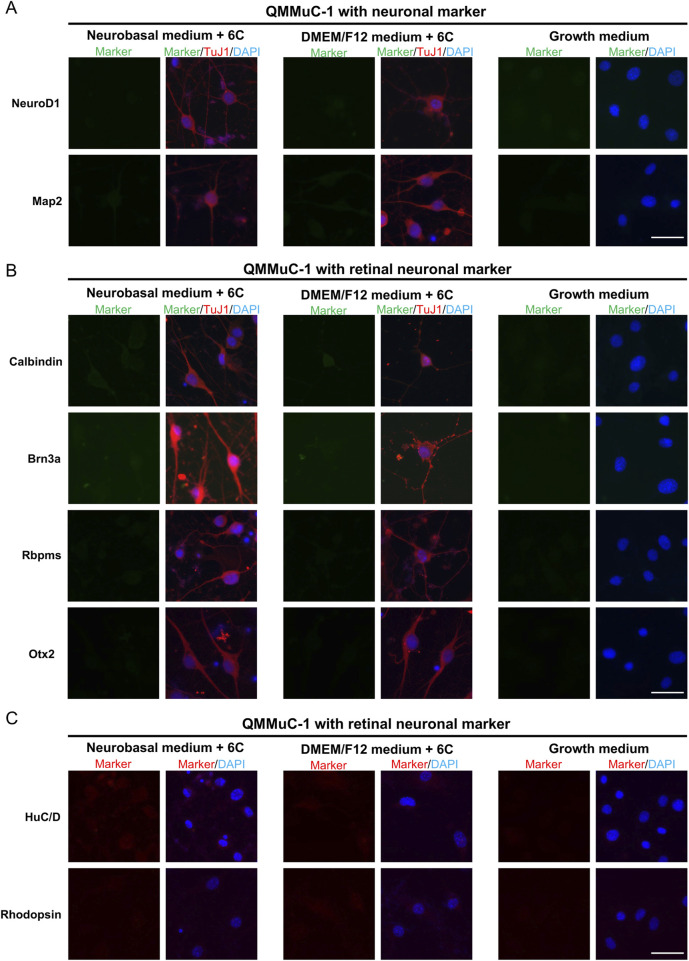
Immunostaining results of neuron-like cells derived from QMMuC-1 cells with various neuronal markers and retinal neuron markers. **(A)** Cells were stained with TuJ1 marker and neuronal markers: NeuroD1 and Map2. Scale Bars, 50 μm. **(B)** Cells were stained with TuJ1 marker and retinal neuron markers: Calbindin (primarily expressed in horizontal cell, as well as in some subtypes of amacrine and retinal ganglion cells); Brn3a and Rbpms (markers of retinal ganglion cells); Otx2 (marker of bipolar and immature photoreceptor). Scale Bars, 50 μm. **(C)** Cells were stained retinal neuron markers: HuC/D (expressed in amacrine and retinal ganglion cells, with transient expression in horizontal cells during development), Rhodopsin (marker of mature rod photoreceptor). Scale Bars, 50 μm. 6C–Medium supplemented with 6 small molecules.

**FIGURE 7:**
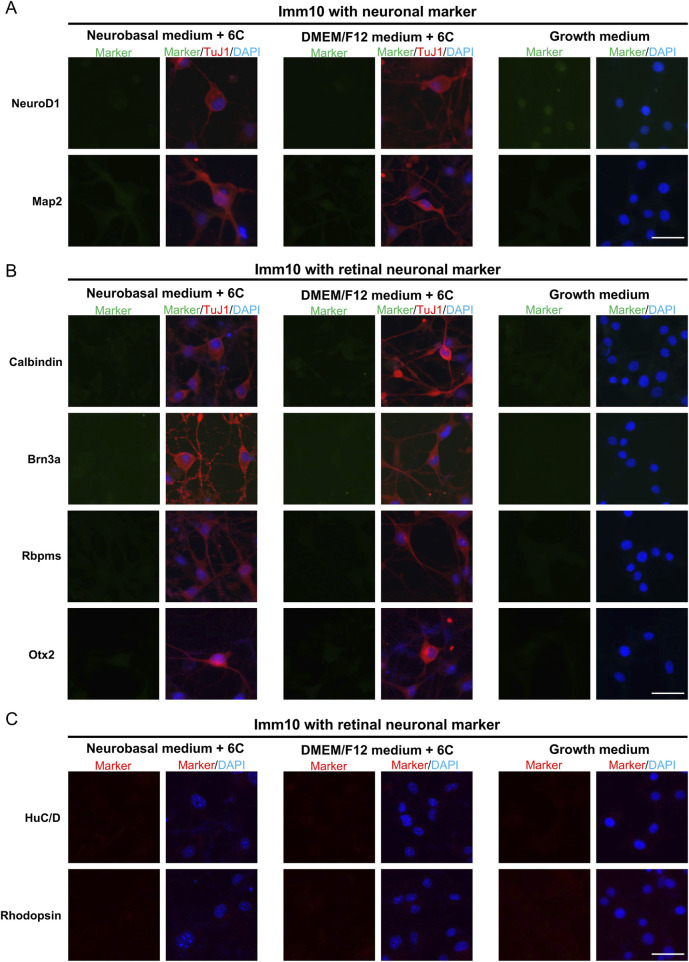
Immunostaining results of neuron-like cells derived from ImM10 cells with various neuronal markers and retinal neuron markers. **(A)** Cells were stained with TuJ1 marker and neuronal markers: NeuroD1 and Map2. Scale Bars, 50 μm. **(B)** Cells were stained with TuJ1 marker and retinal neuron markers: Calbindin (primarily expressed in horizontal cell, as well as in some subtypes of amacrine and retinal ganglion cells); Brn3a and Rbpms (markers of retinal ganglion cells); Otx2 (marker of bipolar and immature photoreceptor). Scale Bars, 50 μm. **(C)** Cells were stained retinal neuron markers: HuC/D (expressed in amacrine and retinal ganglion cells, with transient expression in horizontal cells during development), Rhodopsin (marker of mature rod photoreceptor). Scale Bars, 50 μm. 6C–Medium supplemented with 6 small molecules.

To confirm the neuronal identity of neuron-like cells derived from primary MG, QMMuC-1, and ImM10 cells, we stained these cells with neuronal cell markers (Figures 5A, 6A, 7A) and retinal neuron markers (Figures 5B,C, 6B-C, and 7B-C). None of the TuJ1+ cells in all three cell types were positive for NeuroD1, a member of the basic helix-loop-helix transcription factor family that highly expressed in developing neurons (Cho and Tsai, 2004). In addition, TuJ1+ cells in all three cell types were negative for Map2, a marker of mature neurons, confirming that these cells remained at an immature stage. Interestingly, a few TuJ1+ cells derived from primary MG cells were positive for Calbindin in both types of media (Figure 5B). Moreover, we detected cells expressing the HuC/D marker in the primary MG cells treated with 6 compounds (Figure 5C). Due to limitations with the host antibody, we could not confirm whether these HuC/D+ cells were also TuJ1+; however, because none of the primary MG cells cultured in growth medium were positive for HuC/D, it is likely that these HuC/D+ cells were induced by the 6 chemicals. Calbindin is a calcium-binding protein primarily expressed in horizontal cells, as well as in some subtypes of amacrine and retinal ganglion cells in the mouse retina (Poché et al., 2007; Gu et al., 2016). Similarly, HuC/D is expressed in amacrine and retinal ganglion cells, with transient expression in horizontal cells during development (Ekström and Johansson, 2003). However, primary MG-derived TuJ1+ cells were negative for Brn3a and Rbpms, two markers of retinal ganglion cells. Therefore, these results suggest that a subset of neuron-like cells derived from primary MG cells were likely directed toward amacrine cells or horizontal cells rather than retinal ganglion cells. There was no difference in the percentage of primary MG cells positive for Calbindin/TuJ1 or HuC/D markers on day 7 between Neurobasal and DMEM/F12 media. Surprisingly, unlike primary MG cells, none of the neuron-like cells derived from ImM10 or QMMuC-1 cells expressed Calbindin or HuC/D. All cell types were negative for Otx2, a marker of bipolar cells and immature photoreceptors, as well as Rhodopsin, a marker of mature photoreceptors. Taken together, these results on day 7 indicate that QMMuC-1 and ImM10 cells can be induced into immature TuJ1+/neuron-like cells, similar to primary MG cells. However, unlike primary MG cells, they did not differentiate into amacrine or horizontal cell fates by day 7.

**FIGURE 8:**
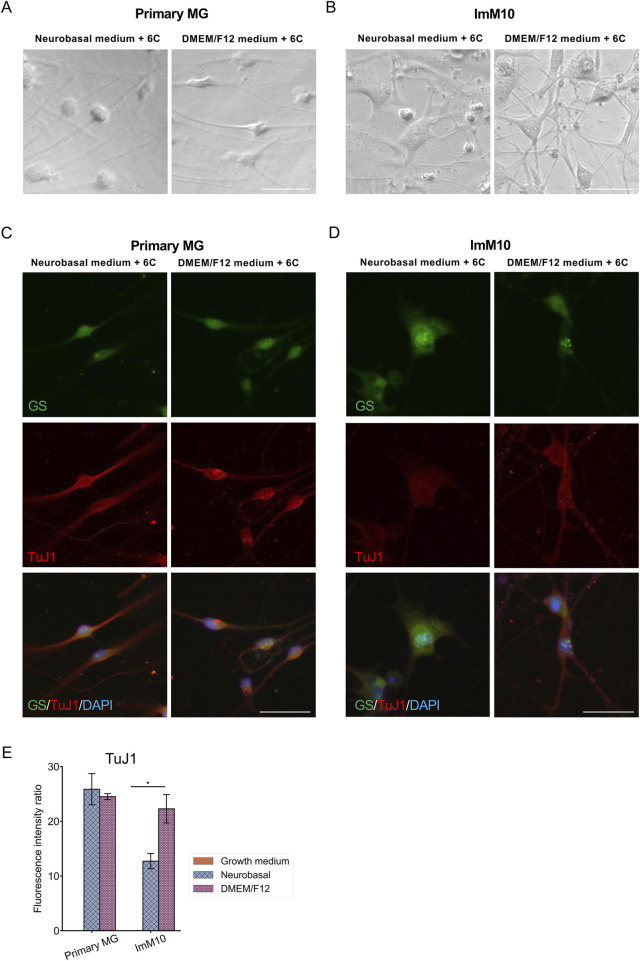
Morphology and immunocytochemistry staining of primary MG and ImM10 cells at day 14 after chemical induction. **(A)** Morphology of primary MG cells at day 14 in Neurobasal and DMEM/F12 media. Scale Bars, 50 μm. **(B)** Morphology of ImM10 cells at day 14 in Neurobasal and DMEM/F12 media. Scale Bars, 50 μm. Primary MG **(C)** and ImM10 cells **(D)** were stained with GS and TuJ1 markers at day 14 post-chemical treatment. Scale Bars, 50 μm **(E)** The fluorescence intensity ratio for TuJ1 marker. 6C–Medium supplemented with 6 small molecules.

Primary MG, QMMuC-1, and ImM10 cells were cultured until day 14 in both Neurobasal and DMEM/F12 media supplemented with 6 small molecules (Figure 8). Interestingly, neuron-like cells derived from primary MG cells survived until day 14 in both media (Figure 8A). In contrast, none of the neuron-like cells derived from QMMuC-1 cells survived until day 14 (data not shown). For ImM10 cells, some neuron-like cells remained in the DMEM/F12 medium on day 14, whereas only cells with MG-like morphology were observed in the Neurobasal medium (Figure 8B). Immunostaining results (Figures 8C– E) showed that the surviving MG-like cells in the Neurobasal medium slightly expressed the TuJ1 marker compared to neuron-like cells, suggesting that these cells could have been induced by the small molecules, but the effects were insufficient to achieve noticeable morphological changes*. In vitro* cells expressing TuJ1 without acquiring a neuron-like shape have also been reported in previous studies (Gascón et al., 2016; Todd et al., 2022). Surprisingly, TuJ1-positive cells derived from primary MG cells in both Neurobasal and DMEM/F12 media, as well as from ImM10 cells in DMEM/F12, expressed GS, a marker of MG. These results indicate that induced neuron-like cells derived from QMMuC-1 cells in both types of media and from ImM10 cells in the Neurobasal medium may require additional supplements to survive beyond day 7. Furthermore, the surviving induced neuron-like cells on day 14 were still in an immature stage.

## 4 Discussion

Our research demonstrates that QMMuC-1 and ImM10 cells share similar morphology and marker profiles with primary MG cells in the growth medium and can be chemically induced into immature neuron-like cells. However, QMMuC-1 and ImM10 cells showed unique characteristics, including higher proliferation rates, distinct survival abilities after chemical induction, and different responses to Neurobasal and DMEM/F12 media supplemented with 6 small molecules.

The QMMuC-1 cell line was generated via spontaneous immortalization, while the ImM10 cell line was established by the conditional overexpression of a proto-oncogene called simian virus 40 large tumor antigen (Otteson and Joseph Phillips, 2010; Augustine et al., 2018). Both cell lines were cultured for up to 50 passages (Otteson and Joseph Phillips, 2010; Augustine et al., 2018). In contrast, primary MG cells stop proliferation and undergo senescence after only 4 to 8 passages (Liu et al., 2017; Pereiro et al., 2020b). Thus, it is not surprising that QMMuC-1 and ImM10 cells have a higher proliferation capacity than primary MG cells in the growth medium. Simian virus 40 large tumor antigen activation in primary mouse embryonic fibroblasts showed a shorter doubling rate and greater viability than in cells cultured under spontaneously immortalized conditions (Amand et al., 2016). Thus, conditional overexpression of simian virus 40 large tumor antigen may induce the ImM10 cell line to acquire a higher capacity to overcome contact inhibition and a faster proliferation rate than the QMMuC-1 cell line.

Immunostaining of cell cultures in the growth medium revealed that primary MG, QMMuC-1 and ImM10 cells expressed GFAP marker. In normal retinas, MG typically show no gene expression or very low gene expression of GFAP (Xue et al., 2006a). After the retinal injury, MG upregulate GFAP expression, indicating their activation and stress (Xue et al., 2006a). A previous study reported that freshly dissociated rabbit Müller glial cells did not express the GFAP gene, but after 2 days of culturing, the cells displayed high levels of GFAP marker expression (Mcgillem et al., 1998). Other studies have also shown that primary MG cells express GFAP markers even when the primary cells are isolated from normal, healthy pups (Augustine et al., 2018; Kittipassorn et al., 2019). In contrast, several research groups have reported that primary MG cells do not express GFAP (Hicks and Courtois, 1990; Limb et al., 2002). The cell isolation protocols used in these studies have many differences, including the type of enzyme used for dissociation and the duration of enzyme incubation. One study suggested that extended soaking during cell isolation in their protocol may reduce the cellular adhesion and the severity of tissue dissociation, resulting in low GFAP gene expression (Hicks and Courtois, 1990). Thus, the differences in GFAP expression in primary cells in previous reports may be due to the differences in the effects of the dissociation step on MG. The W21M MG cell line, an immortalized whale MG, strongly expresses GFAP at passages lower than 10 but starts to reduce GFAP expression after passage 10 (Pereiro et al., 2022). Although in this study, the fluorescence signal intensity of GFAP marker in primary MG was comparable to two immortalized cell lines, primary MG displayed a higher variability. Hence, the lower expression of GFAP in QMMuC-1 and more stable expression of GFAP in ImM10 cells compared to that in primary MG cells may be due to the high passage number of the two immortalized cell lines used in the present study.

The primary MG cells isolated from postnatal mouse pups in the present study expressed nestin and Pax6, consistent with previous studies (Xue et al., 2006b; Pereiro et al., 2020a). MG express nestin and Pax6 during postnatal development but diminish the expression of these markers in the adult stage (Xue et al., 2006b; Kato et al., 2022). Notably, a study has demonstrated that a subset of MG cells continues to express Pax6 during the adult stage (Joly et al., 2011). Several studies have shown that MG upregulate nestin and Pax6 expression following retinal injury (Xue et al., 2006b; Suga et al., 2014; Kato et al., 2022). Both QMMuC-1 and ImM10 cell lines were established from primary MG cells isolated at the postnatal stage; however, QMMuC-1 cells were negative for nestin marker. Interestingly, one study observed that only primary human MG that coexpress nestin and cellular retinaldehyde-binding protein, a MG marker, can become spontaneously immortalized in culture (Lawrence et al., 2007). Thus, it is possible that at early passages of QMMuC-1 cells, these cells express nestin. However, after several passages, these cells may lose nestin expression for unknown reasons.

The concentration of small molecules greatly affects neuronal conversion and the survival rate of induced neurons. A previous study halved the concentration of I-BET151 and ISX9 on day 2 after chemical induction and later added neurotrophins, antioxidants, and a broad-spectrum caspase inhibitor on day 6 to increase the survival of reprogrammed cells (Xia et al., 2021). Another study established a strategy to balance the neuronal conversion efficiency and survival rate by adding small molecules in a stepwise manner, with one group of small molecules inducing neuronal reprogramming, while the other group promoting cell survival and improving reprogramming efficiency (Yang et al., 2019). To reduce cell death, we adjusted the concentrations of I-BET151, ISX9, and CHIR99021 on day 2 after chemical induction. In addition to the small molecule concentration, we found that the volume of the induction medium greatly affected the survival rate of converted neurons. Typically, the volume of the medium has not been reported in direct reprogramming studies. In our preliminary experiments, we observed that a higher volume of induction medium accelerated the direct reprogramming process and caused more cell death, even with the same concentration as in a smaller volume. This observation may explain the differences between our results and those of previous study (Yang et al., 2022). When we first tested the same concentration of small-molecule combinations to convert primary mouse MG cells into neuron-like cells, we observed low cell survival. The previous study utilized 6-well plates, whereas we used 96-well plates, which are more suitable for drug screening and reduce the number of primary MG cells required for experiments (Yang et al., 2022). However, although we improved the cell survival with our treatment, the induced neuron-like cells remained in an immature stage at day 14 after chemical induction. Reduced concentrations of these compounds could potentially lead to a slower neuronal reprogramming process or incomplete downregulation of MG gene expression. Thus, our results highlight the importance of small molecule concentrations in cell survival and reprogramming efficiency.

Although immortalized glial cell lines undergo changes during the immortalization process, many retain a neurogenic capacity that is, to some extent, similar to that of primary glial cells. One study treated primary mouse MG cells with four chemicals and found that this combination upregulated the rhodopsin gene, a specific photoreceptor marker (Fujii et al., 2023). This finding was validated using a rat immortalized MG cell line, which showed a similar upregulation (Fujii et al., 2023). Another study screened 93 small molecules in a spontaneously transformed mouse astrocyte cell line and identified a combination that converted these cells into glutamatergic neurons (Fernandes et al., 2022). In this study, we investigated the neurogenic characteristics of two mouse immortalized MG cell lines, QMMuC-1 and ImM10, in two types of culture media, Neurobasal and DMEM/F12. Previous studies on direct neuronal reprogramming commonly used these media individually or in a 1:1 ratio (Li et al., 2015; Yang et al., 2019; 2022; Ma et al., 2021; Xia et al., 2021; Fernandes et al., 2022; Fujii et al., 2023). Our findings demonstrated that QMMuC-1 and ImM10 cells were chemically converted into neuron-like TuJ1-positive cells similar to primary cells by day 7 in both media. Interestingly, QMMuC-1 cells showed a higher tendency to convert induced neuron-like cells than primary MG and ImM10 cells. Notably, only ISX9 supplementation in Neurobasal medium was able to induce a subset of QMMuC-1 cells to upregulate TuJ1 expression. Nestin is a marker protein for central nervous system stem cells and progenitor cells, and its expression decreases when these cells differentiate into neurons and glial cells (Dahlstrand et al., 1995). The absence of nestin expression in QMMuC-1 cells may facilitate neuronal differentiation following chemical treatments. Similar to primary MG cells, QMMuC-1 and ImM10 cells did not upregulate NeuroD1, a basic helix-loop-helix transcription factor crucial for neuronal generation and cell fate (Cho and Tsai, 2004), nor Map2, a marker of mature neurons after chemical treatment at day 7.

Although QMMuC-1 and ImM10 cells can be induced into immature neuron-like cells similar to primary MG cells, the induced neuronal cells from these two immortalized lines may not differentiate in the same way as primary MG cells. After stimulation with six compounds, a subset of primary MG-derived neuron-like cells appears to differentiate into amacrine or horizontal cell fates, as indicated by HuC/D and calbindin markers. In contrast, neuron-like cells derived from QMMuC-1 and ImM10 cells had not reached the differentiation stage. Another possibility is that TuJ1+ cells derived from QMMuC-1 and ImM10 cells could differentiate in the same direction as primary MG cells, but at a slower rate. Therefore, future work is needed to evaluate cell identity at a more mature stage. However, based on these results, we could at least conclude that QMMuC-1 and ImM10 cells did not respond to the six compounds in the same way as primary MG cells. The high variability of the GFAP marker in primary MG cells suggests that these cells were quite heterogeneous, which may explain the differences in response between the cell types. Firstly, the primary MG cells isolated from postnatal day 2 pups may contain a small subset that is susceptible to neurogenic stimulation, allowing these cells to differentiated into retinal neurons more easily. In contrast, the QMMuC-1 and ImM10 cell lines underwent many passages, thus may have lost these characteristics.

We observed that the induced neuron-like cells derived from QMMuC-1 cells could not be maintained beyond day 7 in either Neurobasal or DMEM/F12 media. In contrast, the induced neuron-like cells derived from ImM10 cells in DMEM/F12 survived until day 14 post-treatment, albeit with a gradual reduction in cell numbers after day 7. Primary MG-derived neuron-like cells, on the other hand, survived until day 14 in both types of media. In the retina, MG support retinal neurons by releasing various factors, including neurotrophins (Tworig and Feller, 2022). Given that only a subset of primary MG cells is converted into induced neuron-like cells, non-converted MG cells may release neurotrophic factors, thereby supporting the survival of converted neuron-like cells. In contrast, immortalized cell lines lose this characteristic during the immortalization process, as exemplified by QMMuC-1’s significantly lower expression of neurotrophic genes than in primary MG cells (Augustine et al., 2018). The study mentioned above, which used a spontaneously transformed mouse astrocyte cell line, also included neurotrophins in the induction medium from day 0, whereas previous studies typically added neurotrophic factors after transitioning to a mature medium (Li et al., 2015; Ma et al., 2021; Fernandes et al., 2022). It remains unclear why neuron-like cells derived from QMMuC-1 and ImM10 exhibit distinct survival abilities, despite both being immortalized cell lines. Differences in the methods used for immortalization may influence their characteristics.

Our results suggest that the two immortalized MG cell lines, QMMuC-1 and ImM10, to some extent, can be used to study the neurogenic characteristics of MG and address the limitations associated with the use of primary MG cells. Using the ImM10 cell line can be particularly beneficial for screening purposes because it carries a GFP reporter gene under the rhodopsin promoter, enabling the robust detection of GFP signals when ImM10 cells are converted into photoreceptor cells (Otteson and Joseph Phillips, 2010). Immortalized cell lines are also particularly valuable for studying the neurogenic capacity of MG in human due to the limited availability of primary human MG cells. However, since the induced neuronal cells from these two immortalized lines did not respond to the six small molecules in the same differentiation direction as primary MG cells, validating key findings from immortalized cells using primary MG cells is essential. In addition, the inclusion of neurotrophic factors or other supportive factors in the induction media is crucial for maintaining the survival of induced neuron-like cells derived from immortalized cells.

While this study provides valuable insights into the neurogenic capacity of immortalized MG cell lines, certain limitations must be acknowledged. Firstly, due to the lack of access to early-passage QMMuC-1 and ImM10 cell lines, we were unable to assess and compare their neurogenetic capacity at early and late passages. This limitation prevents a comprehensive understanding of how genetic or epigenetic drift during immortalization may influence neurogenesis. Future studies focusing on early-passage immortalized MG cell lines will be essential for clarifying the impact of passage number on small-molecule-induced neurogenesis and improving the generalizability of these findings. Secondly, the induced neuronal cells in this study are predominantly at an immature stage. Therefore, further work will be necessary to promote their maturation and evaluate their functional characteristics, such as synaptic connectivity and electrophysiological responses. Finally, while our study highlighted differences in cell survival between the induced neuronal cells derived from primary Müller glia, QMMuC-1 cells, and ImM10 cells in two different media, and suggested potential factors contributing to these differences, we did not perform experiments aimed at improving cell survival. Optimizing conditions to enhance cell survival is a complex task that would require careful adjustments of various factors, and thus, represents an important area for future research.

## 5 Conclusion

In conclusion, we examined and compared the characteristics of primary MG cells and two immortalized mouse MG cell lines, QMMuC-1 and ImM10, in standard growth and neurogenic induction media. In standard growth medium, QMMuC-1 and ImM10 cells showed similar morphology and marker profiles, with differences only in the nestin markers. However, the QMMuC-1 and ImM10 cells exhibited significantly higher proliferation rates. In the chemical induction medium, QMMuC-1 cells and ImM10 cells were converted into immature neuron-like cells, such as primary MG cells with variations in reprogramming efficiency. However, induced neuronal cells derived from QMMuC-1 and ImM10 cells exhibited differences in their differentiation capacity into retinal neurons compared to primary MG cells. Our experiments also showed differences in the cell survival of induced neuron-like cells derived from the three cell types and the effect of the culture medium on direct neuronal reprogramming.

## Data Availability

The raw data supporting the conclusions of this article will be made available by the authors, without undue reservation.

## Abbreviations

Müller glia, MG, glial fibrillary acidic protein, GFAP, 4′,6-diamino-2-phenylindole, DAPI, sex determining region Y (SRY)-box9, Sox9, glutamine synthetase, GS, glutamate aspartate transporter, GLAST, paired box 6, Pax6, class III beta-tubulin, TuJ1, RNA-binding protein with multiple splicing, Rbpms, microtubule-associated protein 2 (Map2).
